# Protective Efficacy of Selenite against Lead-Induced Neurotoxicity in *Caenorhabditis elegans*

**DOI:** 10.1371/journal.pone.0062387

**Published:** 2013-04-26

**Authors:** Wen-Hsuan Li, Yeu-Ching Shi, I-Ling Tseng, Vivian Hsiu-Chuan Liao

**Affiliations:** Department of Bioenvironmental Systems Engineering, National Taiwan University, Taipei, Taiwan; University Zürich, Switzerland

## Abstract

**Background:**

Selenium is an essential micronutrient that has a narrow exposure window between its beneficial and toxic effects. This study investigated the protective potential of selenite (IV) against lead (Pb(II))-induced neurotoxicity in *Caenorhabditis elegans*.

**Principal Findings:**

The results showed that Se(IV) (0.01 µM) pretreatment ameliorated the decline of locomotion behaviors (frequencies of body bends, head thrashes, and reversal ) of *C. elegans* that are damaged by Pb(II) (100 µM) exposure. The intracellular ROS level of *C. elegans* induced by Pb(II) exposure was significantly lowered by Se(IV) supplementation prior to Pb(II) exposure. Finally, Se(IV) protects AFD sensory neurons from Pb(II)-induced toxicity.

**Conclusions:**

Our study suggests that Se(IV) has protective activities against Pb(II)-induced neurotoxicity through its antioxidant property.

## Introduction

Selenium (Se) is an essential trace nutrient that has a narrow exposure window between its beneficial and detrimental effects. Se has numerous beneficial effects on health [1]. Prior studies have shown that Se plays a crucial role in the regulation of glutathione (GSH) and thioredoxin (Trx) systems against oxidative stress and related diseases, such as AIDS progression through GPx and TrxRs [2]. Several studies suggested that neuronal diseases, such as cerebral ischemia, Alzheimer’s disease, and Parkinson’s disease can be attenuated by Se through its antioxidant property [3], [4]. Other neuroprotective effects of Se have been reported at an experimental level in both methamphetamine- and 6-hydroxydopamine-induced toxicities, as well as in positive clinical response during therapy with selenite (Se(IV)) in neurodegenerative diseases [5]. Neural precursor cell death and motor neuron degeneration resulting from trauma can be retarded or inhibited effectively by Se [6]–[8].

Lead (Pb) is a ubiquitous pollutant and a non-essential metal in the ecosystem. Its deleterious effects on brain function was observed 2000 years ago, and forearm paralysis is a typical symptom in lead-exposed workers [9]. Pb is especially toxic to children with decreasing cognitive and increasing behavior disorders, such as aggression and hyperactivity [10], [11]. Other neuropathological effects of chronic Pb exposure include endothelial cell swelling and necrosis in cerebral and cerebellar capillaries, resulting in neuronal cell loss of cerebra, cytoplasmic vacuolization, hyperchromatic cells, chromatolysis, and demyelination of nerve fibers [12]–[14]. The effects of Pb on various neuronal processes and systems, especially the central nervous system (CNS), have been reviewed in detail [15], [16]. In particular, oxidative damage is considered a crucial factor in Pb-induced neurotoxicity, suggesting that Pb induces oxidative stress and/or affects the antioxidant defense system, which can lead to damage of nervous systems through oxidative damage [16].

The nematode *Caenorhabditis elegans* (*C. elegans*) is a vital animal model in the fields of biomedical and environmental toxicology. Moreover, *C. elegans* was established as a model for studying neurotoxicity because it contains 302 neurons; its neuronal lineage is fully described [17], [18]. In addition, neurotransmitter systems, including serotonergic, cholinergic, glutamatergic, and γ-aminobutyric acid (GABA)-ergic synapses and their genetic networks are phylogenetically conserved from nematodes to vertebrates, which allows findings from *C. elegans* to be extrapolated and further confirmed in vertebrate systems [18].

Given Se is an antioxidant and Pb might induce oxidative stress resulting in neurotoxicity, we hypothesize that pretreatment of Se can protect *C. elegans* against the neurotoxicity induced by subsequent Pb exposure. Herein, we investigated the protective potential of Se against Pb-induced neurotoxicity in *C. elegans.* We examined whether Se(IV) can reduce the decline of locomotion behavior in aged animals. In addition, we investigated whether pretreatment of Se(IV) can confer a protective effect against the neurotoxicity induced by subsequent Pb exposure.

## Results

### Se(IV) Ameliorated Declines of Locomotion behavior in Aged Worms

Ageing can have a number of effects of varying severity in the nervous system, including alterations in synaptic efficacy and neuronal death, which may result in behavioral changes or deficits [19]. To examine whether Se(IV) has ameliorative effects on behavior, body bend and head thrash assays were used to assess the locomotory rate of Se(IV) on aged *C. elegans*.

**Figure 1:**
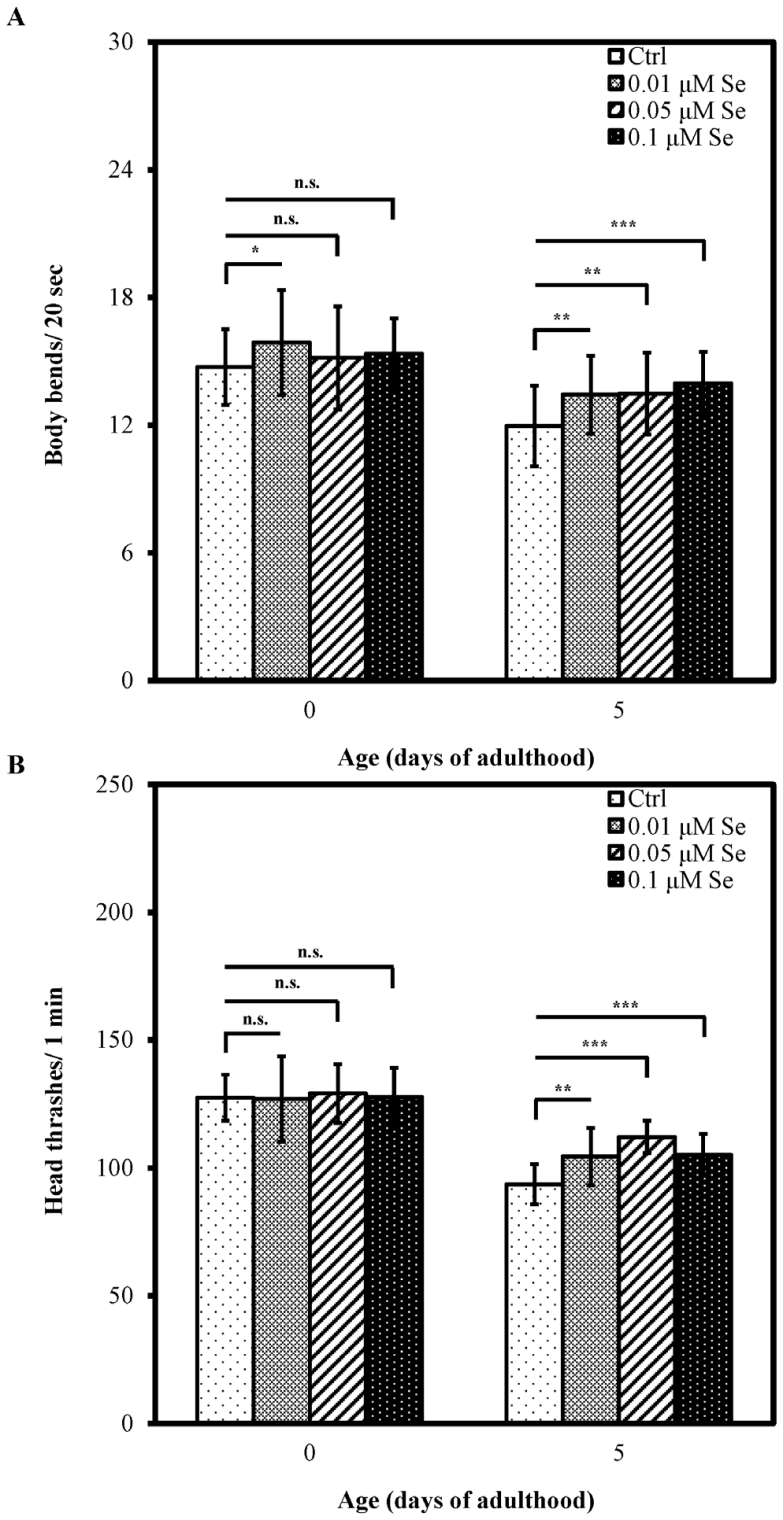
Se(IV) attenuated the declines of locomotion behaviors in aged *C. elegans*. Synchronized wild-type L1 larvae were incubated with various concentrations of Se(IV) (0.01, 0.05, and 0.1 µM) or distilled water as the solvent control at 20°C. The worms at 0 and 5 days old were selected for analysis of the locomotory rate. (A) The number of body bends in 20 s and (B) the number of head thrashes in 1 min. Approximately thirty worms from each treatment at each time point were randomly selected for scoring. Error bars represent the standard error and differences were considered significant at *P*<0.05 (*), *P*<0.01 (**), and *P*<0.001 (***) by one-way ANOVA and LSD post hoc test. n.s., no significant.

We previously showed that 0.01 to 0.1 µM Se(IV) exert ameliorative effects on development and reproduction in *C. elegans* [20]. Thus, we screened 0.01, 0.05, and 0.1 µM Se(IV) to explore the protective action of Se(IV) on locomotion behaviors in aged worms. The results showed slight and non-significant increases between the control and Se(IV)-treated worms in the number of body bends at Day 0 of adulthood (Fig. 1A). However, all Se(IV) treatments significantly ameliorated the aged-related decline of the number of body bends at Day 5 of adulthood (Fig. 1A). Similarly, Se(IV) treatments significantly increased the head thrashes of worms at Day 5 of adulthood (Fig. 1B). Se(IV) ameliorated the decline of locomotion behaviors in aged animals under normal conditions, suggesting that Se(IV) may have protect activity against the aging process in the nervous system.

### Se(IV) Protects the Locomotion behaviors of *C. elegans* against Pb(II)-induced Toxicity

In addition to ameliorating the decline of locomotion behaviors in aged animals, we investigated whether Se(IV) has the potential to protect organisms from chemical-induced neurotoxicity. We selected the Pb(II) neurotoxicant because Pb(II) exposure increases body bends, decreases thermotaxis behaviors, and induces substantial deficits in the structural properties of AFD sensory neurons [21]. The most obvious behavioral output of *C. elegans* is its locomotion which has been used to analyze the response of *C. elegans* to various sensory stimuli. Locomotory behavioral assays including numbers of body bends, head thrashes, and reversal frequency are well established to study neuronal circuits that control behavior [22]. Herein, we selected head thrashing, body bend, and reversal assays to examine the possible protective effects of Se to neurotoxicity on locomotion behaviors induced by Pb(II).

**Figure 2:**
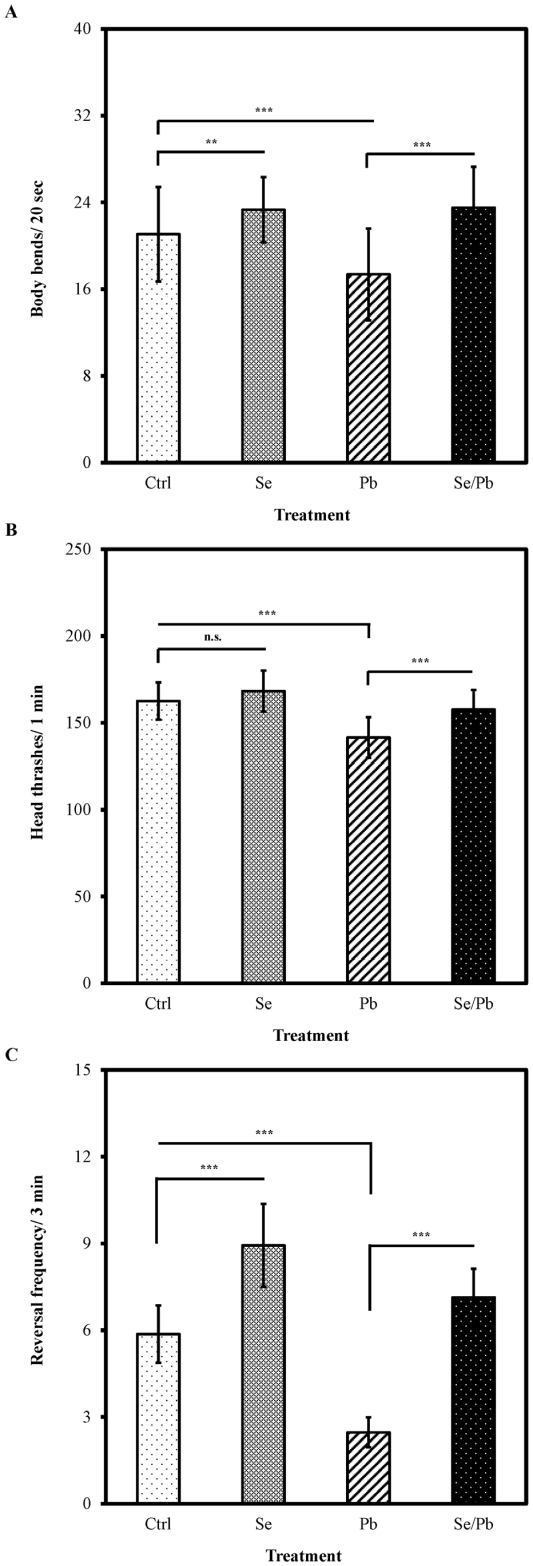
Effects of Se(IV) on locomotion behaviors in ***C. elegans*** under Pb(II)-induced toxicity. Synchronized L1 wild-type larvae were incubated with 0.01 µM of Se(IV) or distilled water as the solvent control for 40 h at 20°C. Subsequently, Se(IV)-pretreated and control young adult worms were divided into two aliquots and treated with or without 100 µM of Pb(II) for 24 h at 20°C. (A) The number of body bends in 20 s, (B) the number of head thrashes in 1 min, and (C) the reversal frequency in 3 min. Approximately thirty worms from each treatment at each time point were randomly selected for scoring. Error bars represent the standard error and differences were considered significant at *P*<0.05 (*), *P*<0.01 (**), and *P*<0.001 (***) by one-way ANOVA and LSD post hoc test. n.s., no significant. “Ctrl”, worms grown on a normal diet; “Se”, worms grown with Se(IV) supplementation; “Pb”, worms grown on a normal diet followed by Pb(II) exposure; “Se/Pb”, worms with Se(IV) pretreatment and followed by Pb(II) exposure.

As shown in Fig. 1, Se(IV) treatment of 0.01 µM is sufficient to ameliorate the decline of locomotion behaviors in aged animals under normal conditions. Therefore, this concentration was selected for the subsequent experiments. In addition, the Pb(II) exposure concentration was selected based on a previous study [21]. The pretreatment using Se(IV) at the concentration of 0.01 µM was performed at the L1-larval stage for 40 h at 20°C, and the following Pb(II) exposure at the concentration of 100 µM was performed for 24 h. As shown in Fig. 2A, Pb(II) treatment significantly decreased the number of body bends of worms compared to those control (*P*<0.001). However, after pretreatment with Se(IV) from L1 for 40 h, the reduction of body bends caused by the subsequent Pb(II) exposure can be prevented in nematodes, compared to those without Se(IV) pretreatment (*P*<0.001) (Fig. 2A), suggesting that Se(IV) can counteract the toxicity induced by Pb(II).

Similarly, without the supplementation of 0.01 µM of Se(IV), a substantial decrease in head thrash occurred in worms exposed to 100 µM of Pb(II), compared to those control (*P*<0.001) (Fig. 2B). Nematodes with Se(IV) pretreatment exhibited significant protection (*P*<0.001) against Pb(II)-induced toxicity on head thrash (Fig. 2B). Although Se(IV) pretreatment cannot fully protect the head thrash behavior against Pb(II) neurotoxicity, as observed in body bend assay, the neuroprotetive effect of Se(IV) is significant (Fig. 2B).

Similarly, without the supplementation of 0.01 µM of Se(IV), a significantly decrease in reversal frequency was observed in worms exposed to 100 µM of Pb(II), compared to those control (*P*<0.001) (Fig. 2C). However, nematodes with Se(IV) pretreatment exhibited significant protection (*P*<0.001) against Pb(II)-induced toxicity on reversal frequency (Fig. 2C). A significant increase in reversal frequency was observed in worms exposed to 0.01 µM of Se(IV), compared to those without Se(IV) control (*P*<0.001) (Fig. 2C), suggesting that Se(IV) has ameliorative effect on reversal frequency to *C. elegans*. Taken together, pretreatment of 0.01 µM of Se(IV) can protect the locomotion behaviors of *C. elegans* against Pb(II)-induced damage.

### Se(IV) Decreases the Intracellular ROS Level in *C. elegan*

This sections explores a mechanism that might explain the manner in which Se(IV) suppresses the decline of locomotion behaviors induced by Pb(II). Pb(II) exposure causes substantial oxidative damage and production of reactive oxygen species (ROS) in *C. elegans,* as described in [21]. Selenium can ameliorate oxidative damage, and cells employ multiple antioxidant mechanisms, including ROS scavenging, to prevent the cellular damage [6], [23], [24]. Therefore, we evaluated the free radical scavenging abilities of Se(IV).

**Figure 3:**
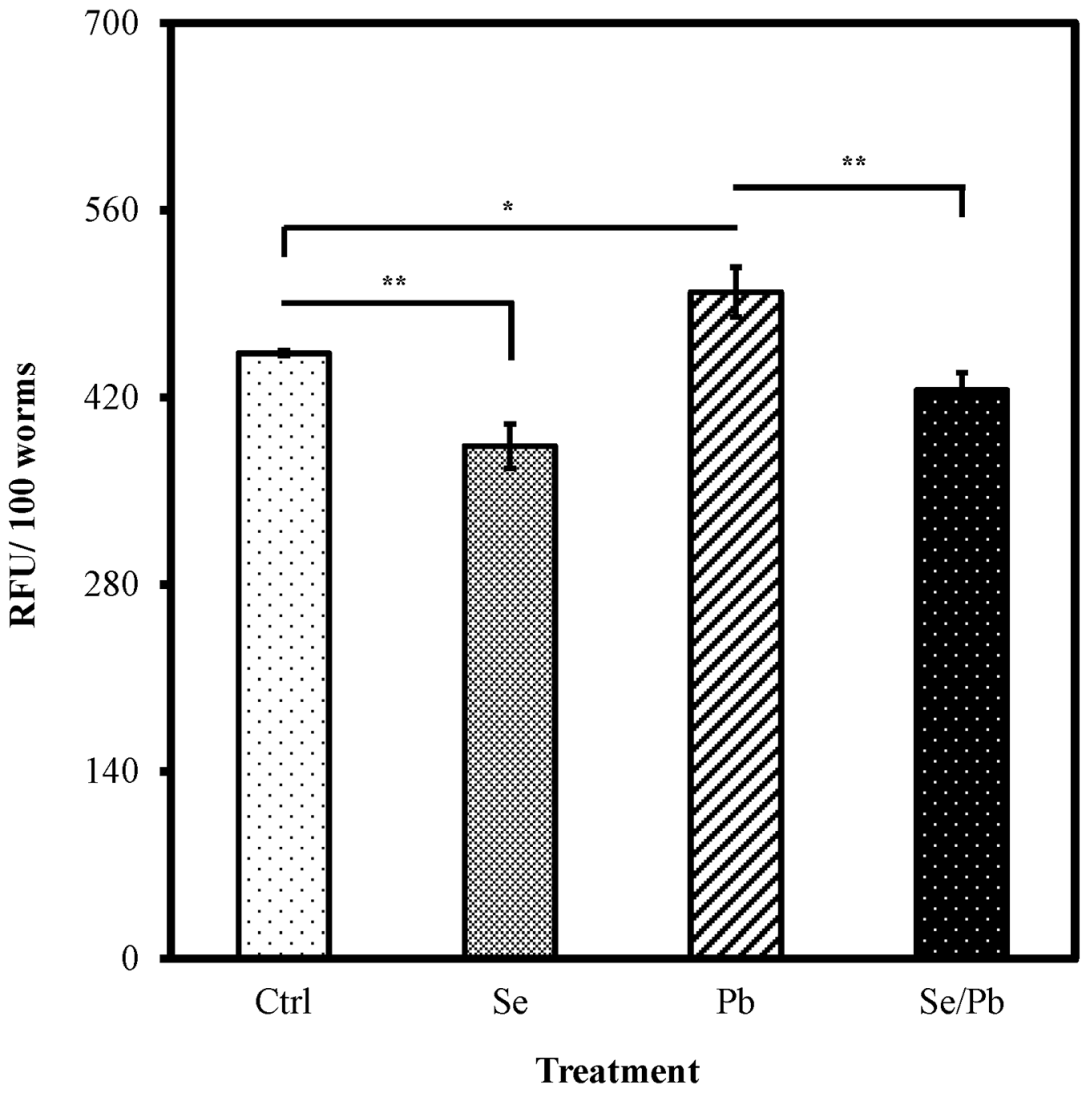
Free radical-scavenging effect of Se(IV) on Pb(II)-induced oxidative stress *in vivo*. Synchronized L1 wild-type larvae were incubated with 0.01 µM of Se(IV) or distilled water as the solvent control for 40 h at 20°C. Subsequently, Se(IV)-pretreated and control young adult worms were divided into two aliquots and treated with or without 100 µM of Pb(II) for 24 h at 20°C. Subsequently, intracellular ROS for adult animals was measured using 2′, 7′-dichlorodihydrofluoroscein diacetate. One hundred worms from each condition were used to analyze the intracellular ROS levels. The results are expressed as relative fluorescence units (RFU) of fluorescence relative to 100 worms. Error bars represent the standard error and differences were considered significant at *P*<0.05 (*), *P*<0.01 (**), and *P*<0.001 (***) by one-way ANOVA and LSD post hoc test. n.s., no significant. “Ctrl”, worms grown on a normal diet; “Se”, worms grown with Se(IV) supplementation; “Pb”, worms grown on a normal diet followed by Pb(II) exposure; “Se/Pb”, worms with Se(IV) pretreatment and followed by Pb(II) exposure.

Wild-type animals were raised from L1 larvae, as described in the locomotion behaviors assays. Subsequently, intracellular ROS for adult animals was measured using 2′,7′-dichlorodihydrofluoroscein diacetate (H_2_DCF-DA). Non-fluorescent DCF-DA is a freely cell-permeable dye that can be readily converted to fluorescent 2′7′-dichlorofluorescein (DCF), because of the interaction with intracellular peroxide (H_2_O_2_). The results showed that 0.01 µM of Se(IV) significantly inhibited the production of ROS in vivo, compared to that in the control (*P*<0.01) (Fig. 3). When worms were exposed to 100 µM of Pb(II), the intracellular ROS level significantly increased compared with that in the control (*P*<0.05) (Fig. 3). Moreover, Se(IV) pretreatment significantly decreased the Pb(II)-evaluated ROS level compared with that for only Pb(II) treatment (*P*<0.01) (Fig. 3). Supplementation of Se(IV) may ameliorate the locomotion behaviors of *C. elegans* by reducing the accumulation of intracellular ROS levels induced by Pb(II), which may damage the nervous system.

### Se(IV) Protects AFD Sensory Neurons from Pb(II)-induced Toxicity

In *C. elegans*, P*gcy-8*::GFP is a specific fluorescent marker that labels the AFD sensory neurons [25]. Therefore, it has been suggested that neuronal damage accompanies significant decreases in the relative sizes of cell body fluorescent puncta and relative fluorescent intensities of cell bodies in AFD neurons [26]. Pb(II) exposure causes significant decreases in the relative intensities of cell bodies in AFD sensory neurons [21].

**Figure 4:**
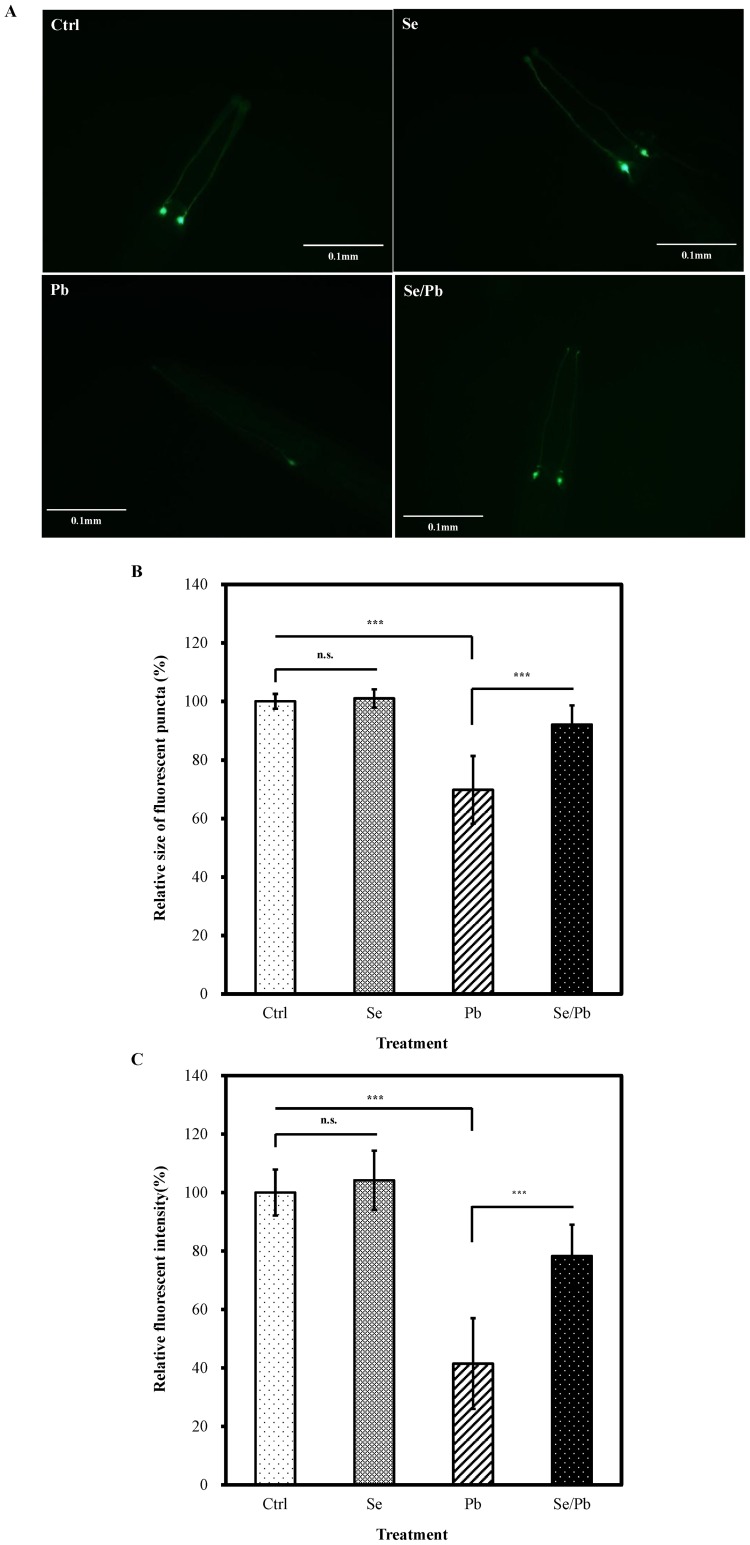
Effects of Se(IV) on AFD sensory neurons by Pb(II) exposure in *C. elegans*. Synchronized L1 P*gcy-8*::GFP transgene larvae were incubated with 0.01 µM of Se(IV) or distilled water as the solvent control for 40 h at 20°C. Subsequently, Se(IV)-pretreated and control young adult worms were treated with 100 µM of Pb(II) for 24 h at 20°C to generate neuronal damage. (A) Representative images of morphological patterns of AFD sensory neurons labeled by P*gcy-8*::GFP. (B) Relative sizes of fluorescent puncta for cell bodies of AFD sensory neurons. (C) Relative fluorescent intensities in cell bodies of AFD sensory neurons. Relative sizes of fluorescent puncta and relative fluorescent intensities were calculated by normalizing to that of control. Approximately thirty worms from each treatment at each time point were randomly selected for analysis. Error bars represent the standard error and differences were considered significant at *P*<0.05 (*), *P*<0.01 (**), and *P*<0.001 (***) by one-way ANOVA and LSD post hoc test. n.s., no significant. “Ctrl”, worms grown on a normal diet; “Se”, worms grown with Se(IV) supplementation; “Pb”, worms grown on a normal diet followed by Pb(II) exposure; “Se/Pb”, worms with Se(IV) pretreatment and followed by Pb(II) exposure.

We investigated the protective capability of Se(IV) on AFD sensory neurons affected by Pb(II) exposure. We examined the relative sizes of cell body fluorescent puncta and relative fluorescent intensities in cell bodies in AFD sensory neurons in worms (P*gcy-8*::GFP) grown with a normal diet compared with those grown with Se(IV) supplementation; the worms were subsequently exposed to Pb(II) incubation. As shown in Figs 4A and 4B, exposure to 100 µM of Pb(II) caused a significant reduction of relative sizes of cell body fluorescent puncta in AFD neurons compared to control (*P*<0.001). However, after pretreatment with Se(IV) from L1 for 40 h, the relative sizes of cell body fluorescent puncta in AFD neurons caused by the subsequent severe Pb(II) exposure can be prevented in nematodes, compared to those without Se(IV) pretreatment (*P*<0.001).

Similarly, without the supplementation of 0.01 µM of Se(IV), a substantial decrease in relative intensities of cell bodies in AFD neurons occurred in worms exposed to 100 µM of Pb(II), compared to those control (*P*<0.001) (Fig. 4C). Nematodes with Se(IV) pretreatment exhibited significant protection (*P*<0.001) against Pb(II)-induced toxicity on relative intensities of cell bodies in AFD neurons (Fig. 4C). Although Se(IV) pretreatment cannot fully protect the Pb(II)-induced toxicity, the protective effect of Se(IV) is significant. Taken together, the result suggests that Se(IV) may protect the AFD sensory neuron cells from Pb(II)-induced toxicity.

### Se(IV) Enhances mRNA Levels of TTX-1, TAX-2, TAX-4, and CEH-14 upon Pb(II) Exposure

We further examined the protective capability of Se(IV) on the expression of genes (*ttx-1, tax-2, tax-4*, and *ceh-14*) required for the differentiation and function of AFD neurons affected by Pb(II) exposure. TTX-1 is a transcription factor that mediates expression of *gcy-8* [25]. The cyclic nucleotide-gated channel α-subunit TAX-4 and β-subunit TAX-2 are thought to function directly in sensory transduction and mediate several sensory behaviors [27], [28]. The LIM homeobox gene *ceh-14* is required for the correct function of the AFD neurons [29]. We examined the changes of mRNA levels of TTX-1, TAX-2, TAX-4, and CEH-14 in Pb(II) exposed and control worms by real-time RT–PCR assays.

**Figure 5:**
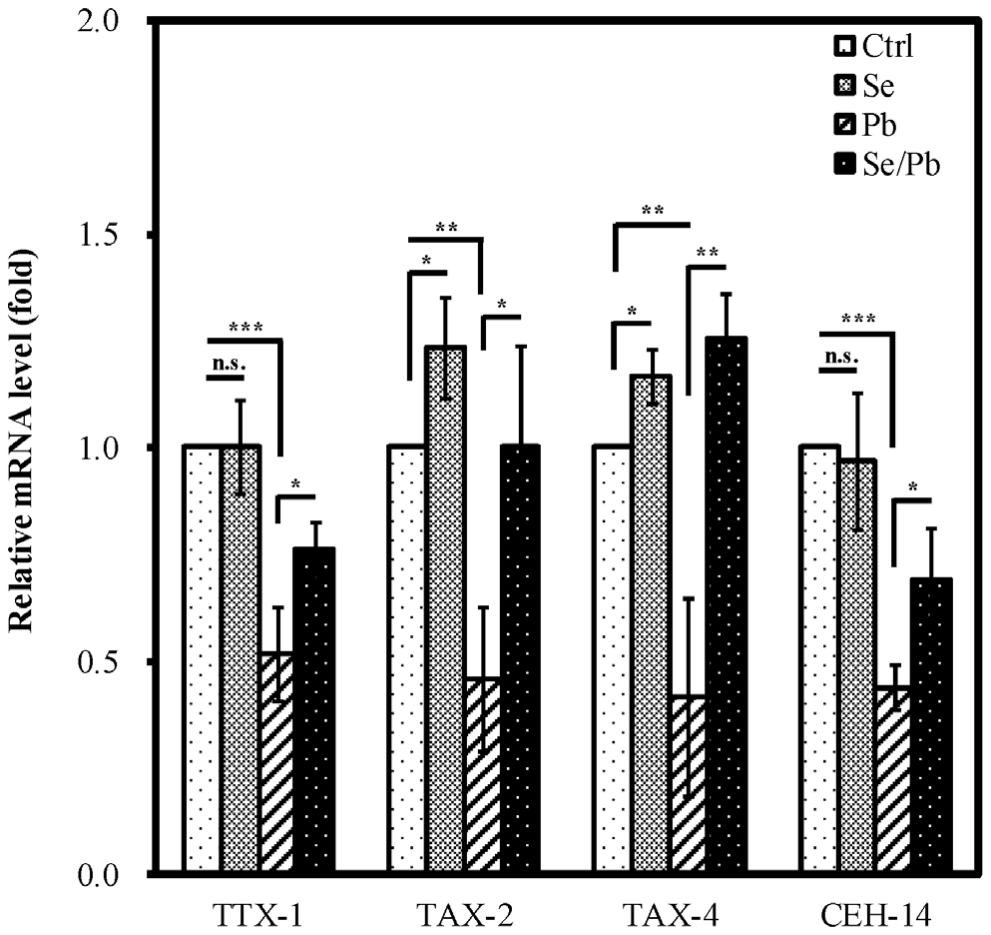
Effects of Se(IV) on the expression of TTX-1, TAX-2, TAX-4, and CEH-14 upon Pb(II) exposure in ***C. elegans***. Synchronized L1 wild-type larvae were incubated with 0.01 µM of Se(IV) or distilled water as the solvent control for 40 h at 20°C. Subsequently, Se(IV)-pretreated and control young adult worms were divided into two aliquots and treated with or without 100 µM of Pb(II) for 24 h at 20°C. Subsequently, the total RNA from adult animals was extracted. mRNA levels of TTX-1, TAX-2, TAX-4, and CEH-14 were determined by quantitative real-time RT-PCR. The mRNA levels were normalized to the expression of ACT-1. The fold change was normalized to that observed in untreated control *C. elegans* samples. The test was performed three times. Error bars represent the standard error and differences were considered significant at *P*<0.05 (*), *P*<0.01 (**), and *P*<0.001 (***) by one-way ANOVA and LSD post hoc test. n.s., no significant. “Ctrl”, worms grown on a normal diet; “Se”, worms grown with Se(IV) supplementation; “Pb”, worms grown on a normal diet followed by Pb(II) exposure; “Se/Pb”, worms with Se(IV) pretreatment and followed by Pb(II) exposure.

When worms were exposed to 100 µM of Pb(II), the mRNA levels of TTX-1 (48%, *P*<0.001), TAX-2 (54%, *P*<0.01), TAX-4 (58%, *P*<0.01), and CEH-14 (56%, *P*<0.001) were significantly decreased compared with that in the control (Fig. 5). Moreover, Se(IV) pretreatment significantly increased the Pb(II)-decreased mRNA levels compared with that for only Pb(II) treatment (Fig. 5). The results suggested that Pb(II) exposure influenced the expression of most genes required for the differentiation and function of AFD neurons and supplementation of Se(IV) may ameliorate such effects.

## Discussion

Selenium is an essential element for human health because it regulates several major metabolic pathways, including immune function and antioxidant defense system [30], [31]. Under normal culture conditions, Se(IV) did not have significant effects on locomotion behaviors, including head thrashes and body bends, at the beginning of adulthood of *C. elegans* (Figs. 1A and 1B). By contrast, Se(IV) pretreatments attenuated the diminutions on locomotion behaviors in 5-day-old adult worms (Figs. 1A and 1B). Moreover, Se(IV) decreases the intracellular ROS level in *C. elegans* (Fig. 3). A crucial feature of aging humans in current society is the increasing events of neurological disorders through elevated oxidative stress [9], [32], [33]. Thus, the antioxidant property of Se may contribute to the age-associated decline of neurobehavior in *C. elegans*.

A significant decrease in body bends, head thrashes, and reversal frequency were observed in wild-type *C. elegans* exposed to 100 µM of Pb(II) compared to Pb(II)-untreated worms (Figs. 2A, 2B, and 2C). These results were consistent with those reported in [21]. The pretreatment of Se(IV) significantly ameliorated the reduction of body bends, head thrashes, and reversal frequency of worms caused by subsequent Pb(II) exposure, compared to that of Se(IV)-untreated worms (Figs. 2A, 2B, and 2C). This suggests that administration of trace amount of Se(IV) can protect the locomotion behaviors of *C. elegans* against Pb(II)-induced damages. Our finding is further supported by mammalian studies, which indicated that Se supplementation has a protective effect when administrated to animals prior to Pb exposure [34].

Oxidative stress is regarded as a main factor in the pathophysiology of various diseases and ageing [35] and it occurs as a result of excessive generation of ROS or diminished antioxidative defense systems. To regulate the overall ROS levels generated from endogenous and/or exogenous sources and protect the cells from stress condition, antioxidant defenses systems and mechanisms are necessary [36]. Several studies have indicated that Pb induces oxidative stress and exerts toxic effects through the disruption of the prooxidant/antioxidant balance [15], [16], [37]. Therefore, oxidative damage is considered a crucial factor in Pb neurotoxicity. We examined the influence of Pb(II) and Se(IV) on the intracellular amount of ROS, and showed that wild-type *C. elegans* grown with Se(IV) (0.01 µM) supplementation exhibited decreased levels of ROS in comparison to those raised on a normal diet (Fig. 3). Moreover, Se(IV) supplementation suppressed the ROS levels after Pb(II) exposure (Fig. 3). This suggests that Se(IV) can alleviate the intracellular level of ROS in *C. elegans* under normal conditions and protect nematodes from Pb(II)-induced oxidative stress.

*C. elegans* senses temperature primarily through the AFD thermosensory neurons, and the response to temperature can be observed as a behavior called thermotaxis on thermal gradients [38]. Three members of guanylyl cyclase genes in *C. elegans* (*gcy-8*, *gcy-18*, and *gcy-23*) and upstream of *tax-4* regulate thermotaxis through the AFD thermosensory neurons [28], [39]. *gcy-8* is expressed exclusively in the AFD thermosensory neurons, where it localizes to sensory endings [25]. Therefore, the relative sizes of cell body fluorescent puncta and relative fluorescent intensities of cell bodies in AFD neurons were used as markers to examine the effects of metal exposure on neuron development [39]. Pb(II) exposure causes significant decreases in the relative intensities of cell bodies in AFD sensory neurons [21]. We further investigated the effects of Se(IV) on neuronal protection against Pb(II) toxicity. When worms were exposed to 100 µM of Pb(II) alone, Pb(II) induced significant decreases in relative intensities and relative sizes of fluorescent puncta of cell bodies in AFD sensory neurons compared with that of the group pretreated with 0.01 µM Se(IV) following Pb(II) exposure (Figs. 4A, 4B, and 4C), suggesting that Se(IV) treatment prior to Pb(II) exposure may protect the AFD sensory neurons from Pb(II)-induced toxicity.

It has been suggested that exposure to higher concentrations of metals (Hg, Cu, Ag, and Cr) resulted in a significant reduction in relative intensities and relative lengths of sensory endings in AFD neurons along with the significant reduction in relative mRNA levels of *ttx-1*, *tax-2*, *tax-4*, and *ceh-14* compared to control [26]. TTX-1 is a transcription factor that mediates expression of *gcy-8* [25]. Pb(II) exposure resulted in approximately 50% reduction in relative mRNA levels of TTX-1 compared to non-exposed control (Fig. 5) which would decrease *gcy-8*::GFP level (Fig. 4). In addition, Pb(II) exposure caused significant reduction of mRNA levels of genes (*tax-2, tax-4*, and *ceh-14*) required for the differentiation and function of AFD neurons. Therefore, Pb(II) exposure at high concentrations (100 µM) might cause toxic effect on the molecular basis for differentiation and function of AFD neurons (Fig. 4). In contrast, nematodes with Se(IV) pretreatment following Pb(II) exposure, the relative mRNA levels of TTX-1, TAX-2, TAX-4, and CEH-14 were significantly increased compared with that for only Pb(II) treatment (Fig. 5). This suggests that Se(IV) pretreatment could effectively protect Pb(II)-induced toxicity to ensure the normal functions of neuron cells.

The antioxidant and toxic properties of Se have been intensively examined in cell culture based mammalian systems but less result was from *in vivo* studies. Morgan *et al.*, (2010) showed that Se(IV) both prevents and induces oxidative stress through a process that involves the GLRX-21 glutaredoxin in *C. elegans* [40]. Recently, Estevez *et al*., (2012) showed that Se-induced oxidative stress leads to neurodegeneration of cholinergic neurons through depletion of glutathione and GLRX-21 glutaredoxin is required for preventing age-related loss of motor neurons in *C. elegans* [41]. Yet, the mechanism through which Se(IV) operates the antioxidant property is not fully understood. In contrast to other animals, TRXR-1, an ortholog of the human enzymatic antioxidant thioredoxin reductase-1, has been reported to be the only selenoprotein in *C. elegans* [42]–[44]. Recent study showed that *trxr-1* null mutant did not show increased sensitivity to oxidative stress after incubation in 2 mM H_2_O_2_ for 2 h [45]. Further investigation will be needed to determine whether Se incorporates into enzymatic antioxidant selenoprotein and the precise mechanisms by which Se(IV) regulates TRXR-1 in *C elegans*. Moreover, the prooxidant mechanisms of Se toxicity will be also required further investigation.

In conclusion, we showed that Se(IV) can attenuate the neurotoxicity that results from Pb(II)-generated oxidative stress. This study provides new evidence for the neuroprotective and antioxidant properties of the mode of actions of Se in organisms.

## Materials and Methods

### Chemicals, *C. elegans* Strains, and Handling Procedures

All chemicals unless otherwise stated were purchased from Sigma-Aldrich (Poole, Dorset, UK). The nematodes used in this study were wild-type N2 and DA1267 (*lin-15*(n765); dEx1267[*lin-15*(+) *gcy-8*::GFP]) labeling the AFD sensory neurons. All *C. elegans* strains and the *Escherichia coli* (*E.Coli*) OP50 strain were obtained from the *Caenorhabditis* Genetics Center (CGC) (University of Minnesota, MN, USA), which is funded by the NIH National Center for Research Resources. Worms were maintained and assayed (unless otherwise stated) at 20°C on nematode growth medium (NGM) agar plates carrying a lawn of *E. coli* OP50 [46]. Synchronization of worm cultures was achieved by hypochlorite treatment of gravid hermaphrodites [47].

### Locomotion behavior Assays

For locomotion behavior assays on aged worms, synchronized L1 wild-type larvae were incubated in liquid S-basal containing *E. coli* OP50 bacteria at 10^9^ cells/ml and various concentrations of Se(IV) (Na_2_SeO_3_) (0.01, 0.05, and 0.1 µM) or distilled water as the control (0 µM) at 20°C. Worms at ages of 0 and 5 days adulthood were selected for analysis of the locomotion behaviors with head thrash frequency and body bend frequency as endpoints.

For Pb(II)-induced locomotion behavior assays, synchronized L1 wild-type larvae were incubated in liquid S-basal containing *E. coli* OP50 bacteria at 10^9^ cells/ml and 0.01 µM Se(IV) or distilled water as the control (0 µM) for 40 h at 20°C. Subsequently, Se(IV)-pretreated and control worms were divided into two aliquots and transferred to K-medium without or with 100 µM of lead (Pb(NO_3_)_2_, Pb(II)) for 24 h at 20°C. A simple line diagram figure displaying the standard method of Se(IV) pretreatment and Pb(II) exposure used in following experiments is presented in supplementary (Figure S1).

The body bend frequency assay was adapted from a previous study [22]. The control and treated nematodes were washed with K-medium 3 times and subsequently transferred onto a second plate and scored for the number of body bends in an interval of 20 s. A body bend was counted as a change in direction of the part of the worm corresponding to the posterior bulb of the pharynx along the Y-axis, with the assumption that the worm was traveling along the X-axis. Thirty nematodes were examined per treatment. The tests were performed at least 3 times.

The head thrash frequency assay was adapted from a previous study [22]. The worms in each treatment were washed with K- medium 3 times. Each worm was transferred into a drop of 60 µl K medium on the top of the agar. After a recovery period of 1 min, the head thrashes were counted for 1 min. A thrash was defined as a change in the direction of bending at the mid body. Thirty nematodes were examined per treatment. The tests were performed at least 3 times.

The reversal assay was adapted from previous studies [22], [48], [49]. The worms from each treatment were washed with K-medium 3 times and then placed onto uncoated NGM plates. Worms were allowed to crawl away from any adherent food at which point they were transferred onto the uncoated NGM plates for reversal counting at 20°C. A period of 1 min elapsed prior to scoring so that worms could recover from the picking. Each worm was observed for 3 min, in which any change from forward to backward movement including omega turns [50], [51] was scored as a reversal. Thirty nematodes were examined per treatment. The tests were performed at least 3 times.

### Measurement of Intracellular Reactive Oxygen Species (ROS)

Synchronized L1 wild-type larvae were incubated in liquid S-basal containing *E. coli* OP50 bacteria at 10^9^ cells/ml and 0.01 µM Se(IV) or distilled water as the control (0 µM) for 40 h at 20°C. Subsequently, Se(IV)-pretreated and control worms were divided into two aliquots and transferred to K-medium without or with 100 µM of lead (Pb(NO_3_)_2_, Pb(II)) for 24 h at 20°C. Subsequently, intracellular ROS in *C. elegans* were measured using 2′,7′-dichlorodihydrofluoroscein diacetate (H_2_DCFDA) (Sigma, St. Louis, MO, USA). One hundred nematodes were broken up using sonication after each treatment, and the worm lysates were collected for the ROS measurement [52]. The worm samples were incubated with H_2_DCFDA (at a final concentration of 50 µM in phosphate buffered saline (PBS) at 37°C in an FLx800 Microplate Fluorescent Reader (Bio-Tek Instruments, Winookski, VT, USA) for quantification of fluorescence with excitation at 485 nm and emission at 530 nm. The samples were read every 20 min for 3 h. A simple line diagram figure displaying the standard method of Se(IV) pretreatment and Pb(II) exposure used in ROS experiments is presented in supplementary (Figure S1).

### Fluorescence Analysis

Synchronized L1 larvae of the DA1267 were incubated in liquid S-basal containing *E. coli* OP50 bacteria at 10^9^ cells/ml and a final concentration of 0.01 µM Se(IV) for 40 h at 20°C. Subsequently, Se(IV)-treated and control DA1267 worms were treated with 100 µM Pb(II) in K-medium at 20°C for 24 h. Fluorescence images of neurons in each treatment group were analyzed. A simple line diagram figure displaying the standard method of Se(IV) pretreatment and Pb(II) exposure used in fluorescence analysis experiments is presented in supplementary (Figure S1).

The relative sizes of fluorescent puncta for cell bodies in AFD neurons were measured as the maximum radius for assayed fluorescent puncta. The relative fluorescence intensity at the cell bodies in AFD neurons was obtained by integrating pixel intensity. At least 30 nematodes were randomly selected worms from each set of experiments were mounted onto microscope slides coated with 2% agarose, anaesthetized with 2% sodium azide, and capped with coverslips. Epifluorescence images were captured using an epifluorescence microscope (Leica, Wetzlar, Germany) with a suitable filter set (excitation at 480±20 nm; emission at 510±20 nm) and a cooled charge coupled device (CCD) camera. The images were photographed and analyzed using Image-Pro Plus software (Media Cybernetics, Bethesda, MD, USA).

### RNA and Real-time Quantitative Reverse-transcription Polymerase Chain Reaction (qRT-PCR) Analysis

Wild-type worms were treated and prepared as described in previous sections. Total RNA from adult worms was isolated using TRIzol according to manufacturer’s instructions (Invitrogen, Carlsbad, CA, USA) and cDNA was synthesized using Super-Script III First-strand synthesis super-Mix for qRT-PCR (Invitrogen). The qRT-PCR was performed on a Step One real-time cycler (Applied Biosystems, Carlsbad, CA, USA) using a SYBR Green qPCR kit (Affymetrix, Inc., Cleveland, Ohio, USA). The qRT-PCR primers were designed for TTX-1 (forward: 5′-TCGGGAACGGACCACATTTA-3′; reverse: 5′-CTTCT GCTGCCTGGCCTTT-3′), TAX-2 (forward: 5′-ACATTTCATCCGTATGGTCGTTT-3′; reverse: 5′-CCGTGGTTTGATTAGCAGCAT-3′), TAX-4 (forward: 5′-TATCCGGATGCAC GAAAGCT-3′; reverse: 5′-GCTTGAGTGCTCCACGATGA-3′), CEH-14 (forward: 5′-CCG GTGGAAGTCCTCAAATC-3′; reverse: 5′-GGTGTCTGCTCTCTGGAGTGAA-3′), and ACT-1 (forward: 5′-GCTGGACGTGATCTTACTGATTACC-3′; reverse: 5′-GTAGCAGAG CTTCTCCTTGATGTC-3′). mRNA levels were normalized to the expression of ACT-1, which encodes the actin isoform. The fold change was normalized to that observed in untreated *C. elegans* samples. The test was performed three times.

### Data Analysis

Statistical analysis was performed using SPSS Statistics 17.0 Software (SPSS, Inc., Chicago, IL., 2008). The results are presented as the mean ± standard errors of mean (SEM). The statistical significance of differences between the populations was determined using one-way ANOVA and LSD post hoc test. Differences were considered significant at *P*<0.05 (see figures).

## Supporting Information

Figure S1 **Line diagram of the standard experimental method.**
(TIF)
